# Designing Light for Emotion: A Neurophysiological Approach to Modeling Affective Responses to the Interplay of Color and Illuminance

**DOI:** 10.3390/biomimetics10100696

**Published:** 2025-10-14

**Authors:** Xuejiao Li, Ruili Wang, Mincheol Whang

**Affiliations:** 1 Department of Emotion Engineering, Sangmyung University, Seoul 03016, Republic of Korea; lixuejiao@hebeu.edu.cn; 2 Jingjinji Spatial Intelligent Perception Collaborative Innovation Center, Hebei University of Engineering, Handan 056009, China; 3 College of Architecture and Art, Hebei University of Engineering, Handan 056009, China; 4 Department of Human-Centered Artificial Intelligence, Sangmyung University, Seoul 03016, Republic of Korea

**Keywords:** illuminance, light color, EEG, ECG, emotion regulation, affective computing, human-centered lighting

## Abstract

As the influence of indoor environments on human emotional regulation and cognitive function becomes increasingly critical in modern society, there is a growing need for intelligent lighting systems that dynamically respond to users’ emotional states. While previous studies have investigated either illuminance or color in isolation, this study concentrates on quantitatively analyzing the interaction of these two key elements on human emotion and cognitive control capabilities. Utilizing electroencephalography (EEG) and electrocardiography (ECG) signals, we measured participants’ physiological responses and subjective emotional assessments in 18 unique lighting conditions, combining six colors and three levels of illuminance. The results confirmed that the interaction between light color and illuminance significantly affects physiological indicators related to emotion regulation. Notably, low-illuminance purple lighting was found to promote positive emotions and inhibit negative ones by increasing frontal alpha asymmetry (FAA) and gamma wave activity. Conversely, low-illuminance environments generally diminished cognitive reappraisal and negative emotion inhibition capabilities. Furthermore, a random forest model integrating time-series data from EEG and ECG predicted emotional valence and arousal with accuracies of 87% and 79%, respectively, demonstrating the validity of multi-modal physiological signal-based emotion prediction. This study provides empirical data and a theoretical foundation for the development of human-centered, emotion-adaptive lighting systems by presenting a quantitative causal model linking lighting, physiological responses, and emotion. These findings also provide a biomimetic perspective by linking lighting-induced physiological responses with emotion regulation, offering a foundation for the development of adaptive lighting systems that emulate natural light–human interactions.

## 1. Introduction

Contemporary individuals allocate more than 90% of their time indoors, where the artificial lighting conditions significantly influence emotional states, cognitive abilities, and general well-being [1]. The emergence of new lifestyles, including remote work and remote learning, has underscored the need for tailored and intelligent indoor settings. Nevertheless, the majority of artificial lighting systems are created statically and fail to consider the fluctuating emotional states and cognitive requirements of users, hence inadequately supporting their emotional regulation capabilities. It is well-established that light can affect human emotions and circadian rhythms via non-imaging routes [2]. Concurrently, researchers have examined the impact of natural light on emotional regulation from several viewpoints. Natural light encompasses the entire spectrum, with varying wavelengths exerting specific regulatory influences on human physiological health and emotions [3]. Recent studies have utilized window devices to control natural light and found that it can significantly augment emotional arousal [4]. The lack of natural light exposure correlates with increased cortisol levels at night, diminished melatonin levels, sadness, and worse sleep quality [5,6]. Natural light, which fluctuates in intensity and color temperature, can regulate human circadian rhythms and enhance emotional stability. Extended exposure to gradually altering natural light can significantly improve individuals’ ability to recuperate from stress and facilitate their shift from negative to positive emotion [7]. Prior studies have predominantly concentrated on static lighting circumstances concerning illuminance and color temperature, neglecting adaptive investigations into emotional regulation through the interplay of these factors, as observed in natural light [8,9,10]. In contrast, the present study aims to analyze the effects of illuminance and light color interactions on emotional regulation. A quantitative model correlating illumination conditions with emotional characteristics was developed by analyzing human brainwave and cardiac data. The originality of this study and its distinctions from prior research are summarized in Table 1. This technology enhances users’ quality of life by manipulating light elements to replicate natural light and quantitatively modulate emotions.

### 1.1. Theory of Emotion Regulation

Emotion regulation is accomplished by modifying emotion responses, including their onset, duration, and expressive style. Emotions can be modulated or transformed, with regulation being the determinant of the ultimate emotional response. Gross established a model of emotion regulation that delineates five essential components: scenario selection, situational change, attentional allocation, cognitive reappraisal, and response modification [12]. Emotional regulation, from an active standpoint, is categorized into spontaneous and non-spontaneous regulation. Individuals frequently employ spontaneous emotional regulation (SER) to manage painful or unpleasant feelings. The predominant emotional regulation mechanisms encompass cognitive reappraisal and emotional suppression. Multiple functional magnetic resonance imaging investigations have demonstrated that the prefrontal cortex has a top-down influence on the amygdala, with this inhibitory action serving as one of the neurological mechanisms mediating SER emotional regulation [13].

Enhanced activity in the left prefrontal cortex or bilateral prefrontal cortex, along with diminished alpha band power in these areas, correlates with elevated or more efficient SER. The FAA values indicate the hierarchical emotional control ability associated with cognitive reappraisal [14]. The parietal brainwave theta/beta ratio (TBR), a significant electrophysiological marker of attentional control, exhibits a negative correlation with the spontaneous emotional modulation of hazardous stimuli [15]. Executive control and emotional inhibition correlate with heightened θ-band power in the medial frontal cortex (FMT), indicating enhanced top-down emotional regulation capabilities [16]. The components linked to positive emotional regulation encompass temporal cortex activity; during the experience of pleasant emotions such as happiness and joy, the temporal cortex is activated [17]. The James–Lange theory posits a bottom-up mechanism for emotion generation, asserting that emotions arise from peripheral physiological responses to external stimuli, including elevated heart rate, altered breathing, and perspiration, which subsequently affect brain perception and produce emotional experiences [18]. The Cannon–Bard theory endorses a top-down approach to emotional regulation, positing that external stimuli influence the body [19]. At the same time, emotions and physiological responses manifest concurrently and independently, with the brain’s central nervous system directly governing emotional experiences and physiological reactions. Consequently, ECG serves as a significant measure of emotional regulation capacity. The vagus nerve’s parasympathetic regulation constitutes the principal descending inhibitory route. Individuals with elevated vagally mediated heart rate variability (vmHRV) levels exhibit superior capacity to regulate negative emotions compared to those with diminished vmHRV levels [20]. Nonetheless, specific studies on emotional regulation have indicated that existing methods are insufficiently applicable in intricate real-world situations. In the domain of lighting, phototherapy is predominantly employed to address depression or cognitive impairment, and there is a paucity of varied research findings about lighting in phototherapy settings.

### 1.2. Lighting and Emotion Regulation Theories

Lighting characteristics exert a systematic influence on emotions through both short-term and long-term mechanisms. In the short term, illuminance primarily shapes sensory comfort and perceptual preference, whereas in the long term, the interactive relationship between illuminance and color temperature plays a critical role in emotional regulation [8]. For instance, prolonged exposure to high illuminance at night suppresses melatonin secretion and increases the risk of depression [21]. Beyond illuminance and color temperature, the role of light color in emotional processes has attracted increasing attention [22]. Previous studies have shown that variations in light spectra not only influence emotional recognition but also induce measurable changes in brain activity and heart rate variability associated with emotional regulation [23].

Veitch and colleagues reported that full-spectrum lighting significantly improves arousal, while research on light color and emotional perception revealed notable differences in valence and arousal between different colors [3]. Plitnick et al. further demonstrated that red and blue light enhanced β power, reduced fatigue, and elicited more positive emotional responses, highlighting that light wavelength can modulate emotion independently of melatonin pathways [24].

Color temperature has also been linked to physiological and psychological outcomes. For example, illuminance levels between 170 lx and 590 lx, as well as color temperatures ranging from 3000 K to 5500 K, substantially affect comfort and relaxation. However, some studies have reported no significant correlation between brainwave activity, heart rate, illuminance, or color temperature [11], underscoring the complexity of these interactions. Using EEG and ECG, Askaripoor et al. found that exposure to both low (2564 K) and high (7343 K) color temperatures, relative to 3730 K, markedly decreased alpha power, indicating changes in autonomic and cortical activity [2]. Similarly, Smolders and colleagues showed that exposure to bright light alleviates psychological fatigue, reduces sleepiness, and enhances physiological alertness, leading to improved task performance [25].

From a biomimetic perspective, the study of light–emotion interactions can be informed by the ways in which humans have evolutionarily adapted to natural light cycles. Variations in daylight simultaneously involve changes in both intensity and spectral composition, which in turn regulate circadian rhythms, mood, and cognitive performance. Emulating these natural mechanisms in artificial lighting design offers a pathway to develop emotion-adaptive illumination systems that align more closely with human physiological and psychological needs. Accordingly, examining the combined effects of illuminance and color provides not only theoretical insight into emotion regulation but also practical implications for biomimetic lighting strategies.

Previous studies have primarily investigated the individual effects of illuminance or color temperature on emotion [8,24]. However, just as natural light affects our emotions through a harmonious interplay of changing color and illuminance, the effects of artificial lighting can only be fully understood by considering the interaction between its color and illuminance. For instance, the same red light might induce arousal and tension at high illuminance but could evoke calmness or intimacy at low illuminance. A research gap remains regarding the complex effects of these combined lighting factors and their neurophysiological mechanisms on specific emotion regulation capacities, such as cognitive reappraisal and emotional suppression.

To address this gap, this study aims to elucidate how the interaction between light color and illuminance affects human emotional states and regulation capacities, using objective physiological indicators from EEG and ECG. We established the following specific hypotheses:

**Hypothesis 1.**  *Low-illuminance environments will decrease overall cognitive resources, thereby degrading physiological indicators associated with cognitive emotion regulation (e.g., frontal midline theta, FMT).*

**Hypothesis 2.**  *Specific combinations of color and illuminance will induce significant changes in specific brainwave patterns related to positive affect and attentional control.*

**Hypothesis 3.**  *A machine learning model integrating multi-modal physiological features from both EEG and ECG will predict the valence and arousal dimensions of emotion with higher accuracy than a single-modality model.*

By establishing 18 systematic lighting environments and comprehensively analyzing the resulting physiological and subjective responses, this study constructs a quantitative model of the lighting–physiology–emotion relationship. Ultimately, we aim to contribute to the development of human-centered, closed-loop affective lighting systems that can perceive a user’s emotional state in real time and provide an optimal lighting environment.

### 1.3. Research Deficiencies and Innovation Opportunities

The advancement of affective computing and artificial intelligence technologies has made intelligent lighting control systems a pivotal focus of study in lighting environment studies in recent years. Dong Keun Kim’s team developed an interactive emotional lighting system that leverages physiological inputs, including electrodermal activity (EDA) and photoplethysmography (PPG), for emotional recognition and control of the lighting system [26]. Fernández-Caballero’s team created an intelligent environmental architecture for the monitoring and regulation of emotions, wherein the system detects emotions via physiological signals and behavioral observation, encompassing facial expressions, EDA, and heart rate variability (HRV), and modulates emotional states by altering three types of light color atmospheres [27]. Dahyun Jung’s team developed a human-centered lighting control system that deduces human cognition and visual tiredness from physiological inputs, thereby enhancing the lighting environment, increasing job efficiency, and reducing weariness [28]. The advancement of these systems offers distinctive insights for the design of human-centered lighting control systems; nonetheless, they possess specific limits: lighting research has not been approached from the perspective of practical lighting control, and varied lighting conditions have not been established as a basis for investigation; the physiological parameters employed for emotional regulation lack sufficient diversity, inadequately elucidating the effects on emotional regulation from the viewpoints of the autonomic and central nervous systems; the research is confined to qualitative studies, devoid of quantitative analysis of lighting parameters, physiological indicators, and emotional experiences; it fails to establish a closed-loop system linking lighting parameter emotional regulation theory and data to intelligent lighting control, thereby lacking the adaptability necessary for human-centered emotional lighting automation regulation.

**Figure 1:**
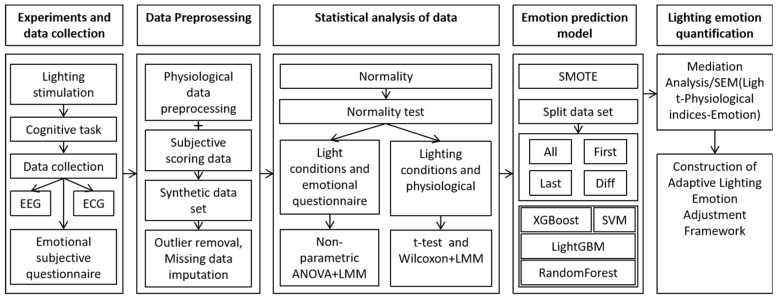
Summary diagram of this study.

This study created 18 lighting conditions consisting of six color temperatures and three levels of illuminance. The study replicated natural light’s ambient conditions by configuring diverse combinations of color temperatures and illuminance levels across the visible light spectrum to examine the effects of lighting environments on human physiological indicators and emotions, thereby addressing the research gap regarding the emotional impact of the interplay between color temperature and illuminance. Experimental data were gathered at various time intervals over 18 illumination conditions, encompassing EEG and ECG data. Physiological data about emotional regulation were retrieved and integrated with self-assessment results derived from the Russell Emotion Theory Model [29] to develop a quantitative model. This methodology addresses the research gap in understanding the quantitative regulation of emotions elicited by illumination, utilizing EEG and ECG time-series analyses. The figure illustrates the specific study methodologies (Figure 1). Develop an adaptive emotional lighting control system to facilitate closed-loop adaptive system research, underpinned by empirical data and theoretical frameworks, offering research trajectories and data support for a biomimetic emotional lighting adaptive regulatory system informed by physiological data.

## 2. Materials and Methods

### 2.1. Lighting Conditions

**Figure 2:**
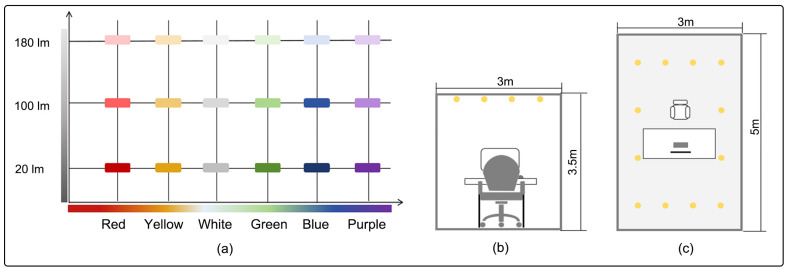
Schematic diagram of the experimental lighting environment: (**a**) A lighting condition matrix consisting of 6 light colors and 3 illuminances; (**b**) Front view of the lighting laboratory; (**c**) Top view of lighting laboratory.

The experiment was conducted in the Lighting Laboratory of the Sensory Architecture Research Institute at Hebei University of Engineering. The lighting laboratory was constructed with dimensions of 3 m × 5 m × 3.5 m. The laboratory’s acoustic and thermal conditions were maintained consistently, featuring non-reflective ceilings and curtains. The dimensions of the table, chairs, and screen conformed to ergonomic standards. The lighting variables comprised an 18-element matrix, consisting of six light colors (white, purple, blue, green, yellow, and red) and three illuminance levels (20 lx, 100 lx, and 180 lx). The light sources were evenly distributed across the ceiling to achieve a visually harmonious lighting effect (Figure 2).

### 2.2. Participants

Thirty-three healthy adults (12 males and 21 females) aged 18 to 36 years (M = 22.5, SD = 3.3) were recruited via email from Hebei University of Engineering. None of the participants reported ocular disorders such as color blindness, cataracts, glaucoma, or other visual impairments. To minimize confounding factors, participants confirmed that they had not consumed caffeine prior to the experiment and had obtained at least eight hours of sleep the preceding night. Written informed consent was obtained from all participants before the study, and the protocol was approved by the Institutional Review Board of Hebei University of Engineering. A priori power analysis (G*Power 3.1; ANOVA: repeated measures, within factors) assuming a medium effect (f = 0.25), α = 0.05, and 1 − β = 0.80 suggested a minimum of *N* = 29; we recruited *N* = 33.

### 2.3. Methodological Approaches

Physiological data were recorded using a 64-channel SAGA system, which allowed for the simultaneous acquisition of EEG and ECG signals. All experiments were conducted in the Lighting Laboratory of Hebei University of Engineering, with one participant tested at a time. After providing informed consent and completing a basic demographic questionnaire, each participant was fitted with a 64-channel EEG cap, connected to the recording device, and monitored by a video camera throughout the experimental session.

Prior to the tasks, participants were introduced to the lighting environment and received a detailed explanation of the experimental procedure. Each participant was then exposed to 18 lighting conditions presented in a randomized order to minimize order effects. At the onset of each condition, participants were allowed to freely scan the environment, after which the EEG recording commenced. Participants first rested quietly for 30 s, followed by an emotional image stimulation task. Upon completion of the task, they were instructed to observe the lighting environment again, remain still for 30 s, and complete the Pleasure–Arousal–Dominance (PAD) questionnaire to rate their subjective experience of the lighting condition [30].

**Figure 3:**
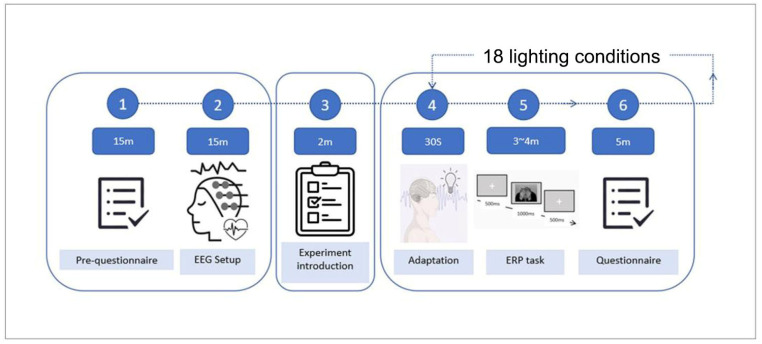
Experiment according to flowchart.

After each lighting condition, participants rested for 5 min and completed a fatigue rating on a 5-point scale. If a score of 3 or higher was reported, an additional 10-min break was provided before proceeding to the next lighting condition (Figure 3).

#### 2.3.1. Subjective Emotional Assessment

The subjective questionnaire was constructed using the PAD, and researchers collected participants’ emotional evaluations on a 1–5 point scale following their exposure to the lighting settings. The PAD is a self-administered questionnaire that measures emotional valence and arousal levels [30]. Furthermore, participants in the experiment were instructed to evaluate 12 pairs of emotional phrases within the Russell Emotional Circle, responding to inquiries such as, “How pleasant does this lighting feel?” These 12 pairs of emotional phrases encompass both positive and negative affect as well as valence and arousal dimensions, capturing a broad range of emotional responses relevant to different lighting environments. The full list of the emotional phrase pairs is provided in Appendix A.2.

In this study, the definitions of valence and arousal were based on Russell’s circumplex model of emotion. Specifically, valence represents the positivity or negativity of an emotion, whereas arousal reflects its intensity or level of activation. Within this two-dimensional model, emotions can be quantified and located according to their valence and arousal coordinates. In affective neuroscience, frontal alpha asymmetry (FAA) is widely recognized as a neurophysiological indicator of affective valence: increased left frontal activity typically corresponds to positive valence, whereas increased right frontal activity indicates negative valence. In addition, oscillatory activities in the parietal and temporal regions, particularly in the alpha, beta, and theta frequency bands, have also been associated with emotional valence.

#### 2.3.2. Physiological Assessment

The EEG assessment of emotion recognition was initially introduced by Walter in 1936, elucidating the correlation between EEG rhythms and cognition through experimental research [31]. Davidson formulated the frontal alpha asymmetry (FAA) theory, suggesting that left frontal activation correlates with positive emotions and appropriate frontal activation correlates with negative emotions. The FAA index is frequently utilized to deduce the valence of emotions and the capacity for emotional regulation. An increased FAA indicates heightened activity in the left frontal lobe, associated with positive emotions [32]. FMT denotes frontal midline theta waves, which predominantly modulate emotions by suppressing executive activities in the prefrontal cortex and reconfiguring negative feelings. Individuals with depression or anxiety often demonstrate impaired FMT function during emotional regulation tasks [33]. TBR represents the ratio of prefrontal theta to beta waves. An elevated TBR value correlates with sustained attention and increased deployment of emotional regulation resources [34]. Accordingly, the study extended beyond the assessment of physiological indicators linked to emotional valence and arousal to include markers of emotional regulation, with particular attention to temporal dynamics within each lighting condition by comparing physiological responses during the initial and later exposure periods. Rosalind W. Picard introduced the concept of affective computing, emphasizing that modeling emotional states through isomorphic physiological signals such as EEG, ECG, and EDA can significantly enhance human–computer interaction and the study of intelligent systems [35]. Zhuozheng Wang’s team employed machine learning techniques to predict emotions utilizing multi-modal physiological data, including EEG and ECG [36]. This study identified time intervals with improved accuracy for emotion prediction under different illumination conditions by analyzing physiological indicators across distinct time periods influenced by these environments.

In ECG evaluation, emotion recognition by ECG data is a prevalent method in contemporary research. Ekman observed in his definition of emotions that particular emotions demonstrate distinct patterns of autonomic nervous system activity, and varying emotional states may arise from the evaluation of the autonomic nervous system [37]. The examination of the temporal dynamic link between ECG data and emotions has been a fundamental focus in emotional research. The heart rate is a multifaceted signal produced by the sympathetic nervous system, parasympathetic nervous system, and physical activity. In high arousal levels, heart rate increases; in low arousal stages, heart rate lowers. Variations in heart rate (HR) indicate the activity level of the sympathetic nervous system (SNS) and function as a bottom-up emotional feedback mechanism. HF mainly indicates parasympathetic activity and exhibits a substantial correlation with vagal tone. A higher heart rate variability (HF) signifies enhanced emotional regulation abilities and improved capability to suppress negative emotions [38]. LF is a composite of the sympathetic and parasympathetic neural systems, correlated with elevated arousal and stress levels. An elevation in LF/HF signifies a proportional augmentation of sympathetic activity and correlates with emotional vigilance [39].

## 3. Data Analysis

### 3.1. Physiological Indicator Data Preprocessing and Feature Extraction

#### 3.1.1. Preparing EEG and ECG Data for Analysis

This research used Python 3.11.7-based technologies, including MNE, Pandas, and NumPy, to preprocess EEG and ECG data for analysis. The preprocessing of EEG and ECG data directly affects the accuracy of the results. During the acquisition of EEG and ECG data, it is inevitable that external environmental factors and instrumentation artifacts introduce irrelevant noise into the recorded signals. The experiment used SAGA equipment for data acquisition, simultaneously recording EEG and ECG data from participants during three time periods: before, during, and after the task. This study focused on the analysis of physiological data collected during two resting states before and after the task in order to derive indicators reflecting the impact of different illumination durations on participants’ emotion-related physiological responses. To ensure the accuracy of the data, multiple signal processing techniques were used to preprocess the EEG and ECG data. The detailed procedure is outlined as follows:

(1) Data files were imported, and raw physiological data were synchronized with event data using timestamps to ensure accurate correlation. The EEG and ECG channels were annotated, and the unnecessary channels were removed.
(2) For EEG data, a 0.5–45 Hz FIR bandpass filter was used to remove low-frequency drift and high-frequency electromyographic artifacts. For ECG data, a 0.5–40 Hz zero-phase FIR bandpass filter was used to remove baseline wander and high-frequency noise. In addition, a 50 Hz notch filter was used to attenuate power line noise further.
(3) Fp1 and Fp2 were assigned as EOG channels, and independent component analysis (ICA) was applied for artifact removal. The signals from Fp1 and Fp2 were summed to enhance blink detection, blink artifacts were manually marked, and the corresponding components were removed before reconstructing the data.

#### 3.1.2. Extracting Features from EEG and ECG

This study primarily aimed to analyze participants’ perceptual, cognitive, and emotional processing under different lighting conditions. Accordingly, EEG feature extraction focused on channels associated with visual perception (occipital: O1, O2), cognitive processing (parietal: P3, P4), and emotional regulation (frontal: F3, F4; temporal: T7, T8). To further investigate the effects of different illumination durations on physiological responses associated with cognition and emotional regulation, features were extracted from two resting intervals: 10 s preceding task onset and 10 s following task completion. The Welch method was employed to calculate the power spectral density (PSD). Power values were calculated by integrating over the θ (4–8 Hz), α (8–13 Hz), β (13–30 Hz), and γ (30–45 Hz) frequency bands, serving as EEG frequency band power metrics for different time intervals. This study analyzed emotion regulatory elements, such as *FMT*, *FAA*, and *TBR* components, using traditional band-specific power measures, to investigate the effects of lighting conditions and duration on emotional regulation capacity. *FMT* represents the θ-band power at the *Fz* channel within the frontal central area. *FAA* was obtained by calculating the difference in alpha band power between the left frontal site (*F*3) and the right frontal site (*F*4):

$$FAA=\log(P_{F4,\alpha })-\log(P_{F3,\alpha })$$

Here, *P_F_*_4,*α*_ and *P_F_*_3,*α*_ represent the alpha wave power at channels *F*4 and *F*3, respectively.

*TBR* was derived by computing the ratio of theta wave power to beta wave power at the frontal central channel *Fz*:

$$TBR=\frac{P_{FZ,\theta }}{P_{FZ,\beta }}$$

*P*_FZ,θ_, and *P*_FZ,β_ denote the *θ*-wave and *β*-wave power of the *Fz* channel, respectively.

For the extraction of ECG Features, identify QRS complexes in the ECG signal and determine the R-wave peak, then calculate the adjacent RR interval (RRi). In the time domain, compute the mean RR interval (MeanRR), standard deviation of RR intervals (SDNN), root mean square of successive RR intervals (RMSSD), count of successive RR intervals differing by at least 50 ms (NN50), and its proportion (pNN50). In the frequency domain, following cubic spline interpolation and resampling of the RRi sequence, the power spectral density is computed using the Welch technique to derive low-frequency power (LF: 0.04–0.15 Hz), high-frequency power (HF: 0.15–0.40 Hz), and their ratio (LF/HF). All ECG characteristics were computed as averages over two temporal intervals: 10 s preceding the task and 10 s following the task.

### 3.2. Statistical Methods for Data Analysis

This study aims to develop a causal quantitative model that elucidates the relationship between light color, illuminance, physiological responses regulating emotion, and self-reported emotional behavior. Prior to data analysis, outliers were identified using the interquartile range (IQR) method and subsequently removed; missing values were imputed, and the data were standardized. The Shapiro–Wilk test was applied to verify that the data did not follow a normal distribution. Consequently, the Scheirer–Ray–Hare test—a non-parametric two-factor analysis of variance—was employed to examine the relationship between lighting conditions and emotional self-assessment, specifically evaluating the effects of light color and illuminance on emotional valence and arousal. A linear mixed-effects regression model (LMM) was used to assess the impact of each lighting variable on valence and arousal, accommodating varying slopes in participants’ responses to lighting. Paired *t*-tests and Wilcoxon signed-rank tests were conducted to evaluate the relationships between illumination conditions and physiological markers. Physiological data were collected during two fixed time intervals: 10 s before and 10 s after the task. To assess the impact of lighting conditions on physiological indicators over time, the analysis focused on the differences between the static physiological measurements recorded 10 s prior to task initiation and those recorded 10 s post-task completion. To identify specific lighting conditions that induced significant changes in physiological indicators, we performed mixed-effects model analyses (LMM) on each highly correlated physiological variable, thereby quantifying the influence of individual lighting variables on these indicators.

### 3.3. Development of an Emotional Quantification Model for Lighting

This research quantitatively analyzes the causal relationships among lighting conditions, physiological indicators, and emotional states using structural equation modeling (SEM). Structural equation modeling is a multivariate statistical framework that integrates measurement models (which describe the relationships between latent variables and observed indicators) with structural models (which specify the directional relationships among latent variables) to estimate latent variables and their interconnections simultaneously [40]. In the model specification, lighting conditions (light color and illuminance) are treated as observed exogenous variables; physiological indicators (EEG characteristics and ECG features) serve as mediating variables; and valence and arousal, derived from self-reported emotional outcomes, are modeled as endogenous latent variables. The model utilizes maximum likelihood estimation with robust standard errors to accommodate non-normal data distributions that diverge from normality, while weighted least squares mean and variance adjusted estimation (WLSMV) is used for model fitting. Given an adequate model fit, standardized path coefficients (β) are reported and interpreted, with their values indicating the direction and strength of the relationships between variables. These standardized path coefficients serve as definitive quantitative metrics for constructing causal models relating lighting-EEG and emotion, as well as lighting, ECG, and emotion.

### 3.4. Development of an Emotion Prediction Model

To examine the variations in emotion prediction derived from physiological data over distinct time intervals, we developed four feature sets utilizing EEG and ECG experimental data: the comprehensive time-series feature dataset, the resting state feature dataset for the initial 10 s under each lighting condition, the resting state feature dataset for the final 10 s prior to the conclusion of the experiment, and the feature dataset emphasizing discrepancies between the first and last 10 s. Non-numeric variables were excluded, retaining only numerical features, and missing values were imputed with the median. Feature scaling was performed to normalize the range of values, and the SMOTE algorithm was employed to oversample minority class samples and address label imbalance. Prior to training, essential features were selected by LASSO, and multiple algorithms, including Random Forest, XGBoost, LightGBM, and SVM, were employed for comparative analysis to determine the most appropriate algorithm for this dataset. These algorithms were selected because they are widely used in affective computing and can handle high-dimensional inputs, capturing complex relationships between physiological features and emotions. Random Forest is a bagging-based ensemble method that is robust to noise, capable of capturing nonlinear relationships, and provides feature importance scores; XGBoost and LightGBM are gradient boosting methods that optimize predictions iteratively but are sensitive to hyperparameters; SVM performs regression in high-dimensional space and is suitable for small datasets, although less flexible for highly nonlinear patterns.

Following model comparison, the Random Forest model achieved the highest prediction accuracy and was therefore selected as the primary model. The Random Forest was configured with 200 decision trees(n_estimators = 200), automatic maximum depth, and a minimum sample split of 2. Feature importance and SHAP analyses were conducted to provide interpretable insights into which physiological features contributed most to emotion prediction, thereby addressing concerns regarding model interpretability.

## 4. Results

### 4.1. Impact of Lighting on Self-Reported Emotional Responses

**Figure 4:**
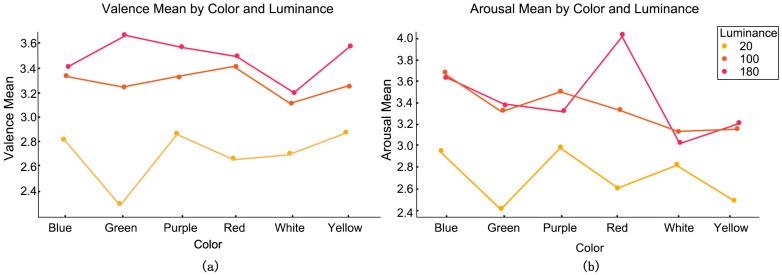
Emotional rating trend chart under different lighting conditions: (**a**) Valence score trend chart under different lighting conditions; (**b**) Arousal score trend chart under different lighting conditions.

The Scheirer–Ray–Hare test, a nonparametric two-factor analysis of variance, was employed to examine the impact of lighting color and illuminance conditions on self-reported emotional outcomes. The findings demonstrated that illuminance (*p* < 0.01) had a substantial impact on valence, while both illuminance (*p* < 0.01) and light color (*p* < 0.01) significantly influenced arousal, as illustrated in Table 2. Green 180 lx demonstrated the highest valence intensity (Mean = 3.66, SD = 0.28), whereas Green 20 lx displayed the lowest valence intensity (Mean = 2.29, SD = 0.31). Red 180 lx demonstrated a greater arousal level (Mean = 4.04, SD = 0.30), whereas Green 20 lx displayed the lowest arousal intensity (Mean = 2.41, SD = 0.32), as illustrated in the image (Figure 4).

A linear mixed model (LMM) was employed to analyze variations in emotional evaluations across distinct lighting situations, incorporating “subject” as a random variable and “light color × illuminance” as a fixed factor. The findings indicated that illuminance exerted a significant primary influence on the valence dimension compared to the baseline condition (white light, 100 lx), with 20 lx illuminance markedly decreasing valence ratings (β = −0.417, *p* = 0.047). A slight interaction between light color and illuminance was seen, with the green light at 20 lx condition exhibiting a marginally significant negative impact on valence scores (β = −0.542, *p* = 0.068). The arousal dimension revealed a significant main effect of light color, with blue light markedly enhancing arousal (β = 0.542, *p* = 0.030). The interaction between illuminance and light color was significant in the arousal model, with red light at 180 lx demonstrating a notable positive effect on arousal (β = 0.792, *p* = 0.025).

In summary, these results from the Linear Mixed Model confirm that the interaction between light color and illuminance is a significant factor, particularly in modulating the arousal dimension of emotional response.

### 4.2. Effects of Lighting Conditions on Physiological Indicators

**Figure 5:**
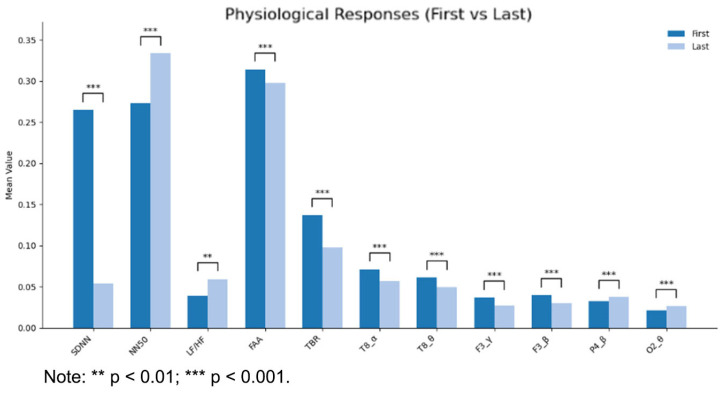
Diagram showing the correlation between physiological indicators at different times and lighting conditions.

This study employed paired *t*-tests and Wilcoxon signed-rank tests to analyze differences in physiological indicators between two time points—10 s prior to task initiation and 10 s post-task completion—under varying lighting conditions. Bonferroni correction was applied to control for the family-wise error rate resulting from multiple comparisons. The results demonstrated statistically significant differences (*p* < 0.01) in specific frequency bands of the T8, F3, O2, and P4 channels, as well as in certain emotion regulation parameters, across different lighting environments. Comparative analysis of physiological indicator changes between the two temporal points under different lighting conditions revealed that alterations in lighting environments could effectively modulate EEG and ECG data, with significant variations observed across the examined time periods. The impact of lighting on ECG parameters was primarily concentrated in three variables: SDNN, NN50, and the LF/HF ratio. These ECG metrics are conventionally regarded as primary indicators of emotional arousal in emotion-related research [41,42]. The effects on EEG indicators predominantly involved parameters associated with emotional regulation, as well as specific frequency bands in the parietal and temporal regions [43,44]. In emotion research, scholars have identified the frontal lobe as a critical region for emotion perception and processing, with the left frontal lobe serving as the principal area for emotion valence regulation and the temporal lobe playing a significant role in positive emotion processing [45,46]. The findings of this study suggest that variations in lighting conditions can influence emotional arousal and valence through modulation of physiological indicators (Figure 5).

Further linear mixed model (LMM) analysis revealed correlations between specific lighting parameters and variations in physiological indicators, as presented in Table 3. Illuminance and light color influenced changes in various physiological parameters, particularly those related to emotional regulation, exhibiting significant regulatory effects.

The low-illuminance condition of 20 lx (β = −0.043, *p* = 0.035) was negatively correlated with FMT, suggesting that reduced illuminance may weaken prefrontal control recruitment during cognitive reappraisal and contribute to more negative emotion evaluations.

High-illuminance green light (β = 0.076, *p* = 0.009) and low-illuminance green light (β = 0.071, *p* = 0.015) demonstrated positive correlations with FMT, while high-illuminance green light (β = 0.520, *p* = 0.040) significantly increased TBR. These findings suggest that green light may facilitate prefrontal engagement during cognitive reappraisal and enhance conflict-suppression mechanisms. This interpretation is consistent with EEG evidence of increased prefrontal phase consistency under green stimulation, as well as broader findings that exposure to green environments can support emotion regulation processes [47].

Purple 20 lx light significantly enhanced FMT (β = 0.589, *p* < 0.001) and FAA (β = 0.049, *p* < 0.001), while also increasing left frontal gamma activity (β = 0.056, *p* = 0.028)—neural markers linked to positive emotion regulation and cognitive control. These findings align with previous research showing increased prefrontal gamma oscillations during meditation and cognitive reappraisal, suggesting that purple light may promote stable positive emotion [48]. Additionally, purple light exposure was positively correlated with the LF/HF ratio (β = 0.712, *p* = 0.044), indicating moderate sympathetic activation and increased vigilance. Overall, our results suggest that Purple 20 lx light supports positive emotional regulation and attentional stability, while also creating a calming yet engaging environment that is distinct from the arousal effects of blue or red light.

Numerous studies have consistently demonstrated that blue light activates intrinsically photosensitive retinal ganglion cells (ipRGCs), thereby enhancing alertness and cognitive performance [49]. Our findings align with this evidence, showing that blue light is positively associated with increased vigilance, as indicated by higher LF/HF ratios. Beyond confirming previous results, we provide novel physiological evidence from autonomic nervous system activity: both blue light (β = 0.115, *p* = 0.004) and low-illuminance blue light at 20 lx (β = 0.121, *p* = 0.031) were positively correlated with LF, suggesting stronger rhythmic regulation of the autonomic system. This may indicate that blue light not only facilitates arousal and attentional control but also contributes to more flexible emotional regulation through enhanced LF dynamics.

In contrast to blue light, red light exhibited a distinct autonomic signature. Exposure to red light showed a positive correlation with LF (β = 0.085, *p* = 0.031), indicating improved rhythmic control of the autonomic nervous system [50,51]. Under high-illuminance red light, HF significantly decreased (β = −0.152, *p* = 0.041), reflecting reduced parasympathetic activity and a shift toward sympathetic dominance. Additionally, SDNN significantly increased (β = 0.937, *p* = 0.023), suggesting enhanced overall heart rate variability, which may be associated with increased emotional variability. These findings suggest that blue light primarily enhances arousal and attentional regulation, whereas red light, particularly at higher illuminance levels, may induce heightened emotional arousal and variability. This supports the notion that different light spectra exert distinct effects on autonomic and emotional regulation.

### 4.3. Causal Model of Lighting, Physiology, and Emotion

**Figure 6:**
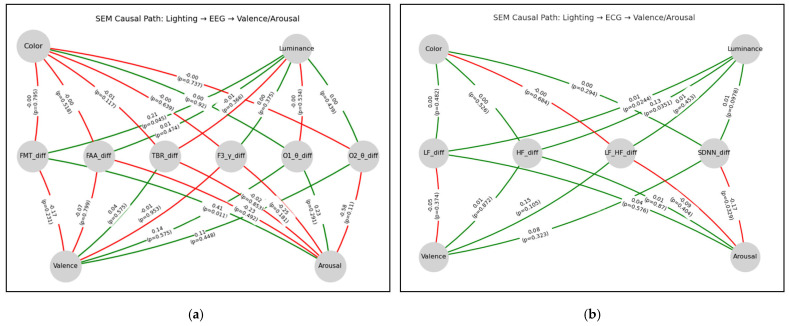
Lighting−Physiological Indicators−Emotional Structural Equation Model: (**a**) SEM Causal Path: Lighting−EEG−Emotion; (**b**) SEM Causal Path: Lighting–ECG–Emotion.

In the EEG model (Figure 6a), the SEM analysis revealed that the proposed causal model provided an adequate fit to the data (CFI = 0.91, TLI = 0.90, RMSEA = 0.06). Illuminance had a direct effect on frontal alpha asymmetry (FMT) (β = 0.21, *p* < 0.05), which in turn significantly influenced arousal (β = 0.41, *p* < 0.05), suggesting a decline in emotion regulation capacity. In the ECG model (Figure 6b), the SEM analysis similarly showed that the proposed causal model achieved an adequate fit (CFI = 0.94, TLI = 0.91, RMSEA = 0.08), Illuminance significantly impacted the HF ratio (β = 0.13, *p* < 0.05), and SDNN was directly linked to arousal (β = −0.17, *p* < 0.05).

### 4.4. Multi-Modal Emotion Regulation Timing Prediction Model Based on EEG and ECG Data

**Figure 7:**
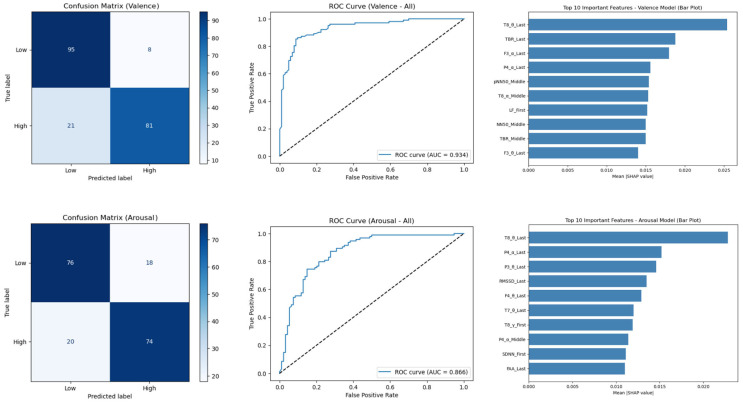
Valence and Arousal Emotion Prediction Model results.

Evaluation of feature sets from different time segments showed that the resting-state data from the first 10 s yielded the best performance. The Random Forest model achieved an accuracy of 89% for valence and 79% for arousal (Table 3). SHAP plot analysis (Figure 7) not only identified key features—temporal lobes (T8) and TBR for valence, and T8 activity and HRV indicators for arousal—but also quantified their individual contributions to each prediction. This provides an interpretable mapping between physiological signals and emotion dimensions, demonstrating how Random Forest predictions are informed by specific EEG and ECG features. While LightGBM, XGBoost, and SVM achieved moderate prediction performance (Table A1), these models either lacked direct feature importance measures or required additional post-hoc interpretability methods. In contrast, the Random Forest model inherently provides feature importance metrics, and the SHAP analysis further enhanced the interpretability of its predictions. The top five features, according to SHAP values, accounted for over 65% of the predictive power for valence and 70% for arousal, highlighting the physiological contributors in the model (Table 4).

## 5. Discussion

This study demonstrated through multi-modal physiological signals that light color and illuminance exert a complex influence on human emotion and emotion regulation capacity, not only independently but also through their interaction. Building on this, we employed structural equation modeling (SEM) to develop a quantitative model linking physiological signals to emotion regulation capacity, providing a systematic framework to quantify how specific lighting conditions modulate emotional responses and regulatory processes. Unlike previous studies that investigated either illuminance or color in isolation, the present work systematically examined their interaction, thereby providing novel evidence that lighting should be considered as a multidimensional and dynamic factor in affective regulation. By integrating EEG and ECG measurements, the present work simultaneously captured responses from both the central nervous system (CNS) and the autonomic nervous system (ANS), offering a more comprehensive account of human reactions to lighting conditions. This dual approach provides novel insights, suggesting that lighting design can be quantitatively interpreted not only as a matter of perceptual preference but also through neurophysiological mechanisms.

As predicted in Hypothesis 1, lower illuminance levels were generally associated with a decline in emotion regulation capacity. Specifically, the decrease in FMT activity, which is related to cognitive reappraisal, implies that low-light environments may reduce attentional or cognitive resources, thereby impairing the ability to reinterpret negative emotions positively. This suggests that illuminance settings in nighttime work or study environments should be carefully considered for their impact on an individual’s stress management capabilities [52].

One of the most notable findings is that purple 20 lx light significantly enhances positive emotions. This was confirmed by an increase in the FAA index, indicating greater left frontal lobe activation, and an increase in left frontal gamma wave activity, which is associated with positive emotion regulation and the inhibition of negative emotions [53,54,55]. Psychologically, the color purple is often associated with creativity and mystique. It is plausible that these characteristics, combined with the calm and stable environment of low illuminance, induce an intrinsic and stable positive emotional state without causing excessive arousal. This finding transcends the conventional wisdom that “brighter is better,” showing that a delicate combination of specific colors and illuminances can play a crucial role in emotion regulation.

Overall, we found that low illuminance generally impairs emotional regulation ability; however, this effect varies significantly depending on the interaction between illuminance and light color. For instance, low-illuminance blue and purple light both exhibit positive effects on emotional regulation: blue light may enhance emotional regulation by modulating heart rate variability, while purple light may promote emotional control by increasing prefrontal gamma oscillations associated with meditation [56]. Additionally, low-illuminance green light effectively improves cognitive reappraisal ability, thereby contributing to emotional stability. These findings suggest that the combination of illuminance and light color regulates emotions through dual pathways involving central nervous system mechanisms (EEG) and autonomic nervous system mechanisms (HRV). Unlike previous studies that primarily focused on the individual effects of light color or illuminance, our results demonstrate that the interaction between light color and illuminance not only shapes emotional experiences but also influences the neural and autonomic mechanisms underlying emotional regulation, underscoring the importance of investigating their interactive effects.

The machine learning-based emotion prediction model is another key contribution of this study. The high prediction accuracy achieved by integrating EEG and ECG data demonstrates that a comprehensive understanding of complex human emotional states requires consideration of both the cognitive processing of the central nervous system and the physical responses of the autonomic nervous system. The SHAP plot analysis, which identified the importance of the temporal lobe and TBR activity for valence (related to cognitive control processing) and parietal lobe activity and cardiac responses for arousal (related to top-down control mechanisms and alertness), provides practical guidelines for which physiological indicators to focus on in the development of future emotion recognition systems. While the machine learning models employed in this study achieved high predictive performance, their practical adoption requires greater explainability. Incorporating interpretability tools such as SHAP analysis or feature importance visualization could help identify the relative contributions of specific lighting parameters to affective outcomes, thereby strengthening the reliability of these models for clinical and industrial applications.

**Figure 8:**
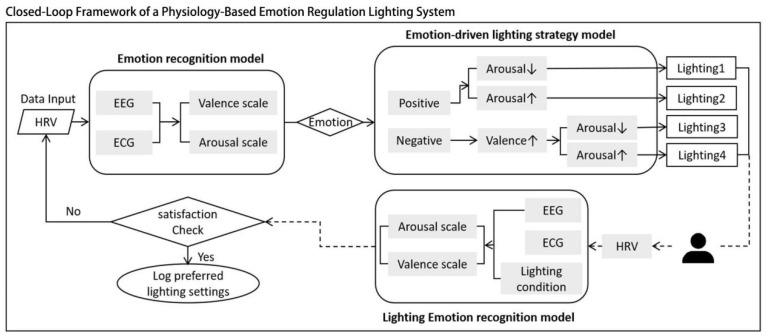
Closed-loop framework of a physiology-based emotion-regulation lighting system.

The results of this study support the feasibility of the closed-loop emotion-regulating lighting system proposed in Figure 8. By monitoring a user’s EEG and ECG data in real time with wearable sensors, an emotion prediction model can identify the current emotional state (e.g., stress, low concentration). Based on this information, the system can automatically execute a predefined lighting strategy (e.g., switching to low-illuminance purple lighting to alleviate stress) to regulate the user’s emotion in a positive direction.

Nevertheless, this study has several limitations. The relatively small sample size may constrain the generalizability of the findings. Although the gender distribution was unbalanced, the results establish a valuable pilot-scale foundation. Future work should employ larger, demographically diverse cohorts to strengthen the external validity. While the PAD scale effectively captured subjective affect, complementary use of additional instruments such as the Self-Assessment Manikin (SAM) or PANAS could enrich the multidimensional understanding of emotional responses in future studies. Because the present study employed short-term exposures in a controlled laboratory setting, the ecological validity of the results remains limited. Several prior studies have demonstrated that short-term light exposure can produce measurable changes in subjective emotional affect and emotional regulation markers. For instance, Chen found that the effects of short-term light exposure on subjective affect and comfort depend on the lighting time of day, while brief white light exposure at different times of day significantly modulated affect and comfort ratings in healthy adults [57]. Moreover, fMRI studies have shown that even very short exposures to blue light can significantly alter activity in emotion-related brain regions such as the amygdala and temporal cortex, indicating that the spectral quality of light can rapidly influence emotional regulation processing [58,59]. Additionally, studies comparing illuminance and color temperature in short exposure paradigms have shown significant effects on emotion perception and neural responses, indicating that markers such as FAA and spectral EEG components respond acutely to lighting changes [60]. Taken together, these findings suggest that short-term exposure studies provide a foundational understanding for designing long-term interventions and thus motivate longitudinal investigations in naturalistic environments, such as offices, hospitals, and classrooms, are warranted to examine whether the observed effects persist over time. Furthermore, while the applied machine learning models demonstrated robust predictive performance, their interpretability was enhanced through the use of SHAP values, which provided insights into the contribution of specific EEG and ECG features. Nevertheless, SHAP remains a post-hoc method, and future studies could benefit from integrating complementary explainable AI techniques and testing generalizability across larger and more diverse cohorts to further improve model transparency and trustworthiness in applied settings.

## 6. Conclusions

In summary, this study demonstrated that the interaction of lighting color and illuminance significantly affects human affective and physiological responses, and that these effects can be reliably predicted using EEG and ECG machine learning models. This study not only advances theoretical understanding but also suggests practical applications for human-centered lighting. For example, low-illuminance purple lighting may support stress recovery in healthcare environments, while high-illuminance blue lighting may enhance concentration in workplace settings.

Despite limitations related to sample size and short-term exposure, the present work establishes a strong foundation for future longitudinal and ecologically valid studies, which in turn may incorporate multimodal physiological signals such as electrodermal activity, respiration, and skin temperature.

Ultimately, by bridging neuroscience, physiology, and environmental design, this research contributes toward the development of smart lighting systems and international standards (e.g., ISO/IEC), positioning illumination as not only an aesthetic or functional element but also a core technology for promoting human emotional and physiological well-being.

## Figures and Tables

**Table 1:** Comparison of previous and proposed methods.

Lighting Variables	Methods	Weaknesses
Six lighting colors [10].	ANOVA was performed to compare emotional states and lighting variables.	Luminance was not considered, and there were no quantitative analyses.
Illuminance and color temperature [11].	ANOVA, post-hoc test, and correlation analyses examined relationships between emotional and physiological variables.	Light color was not considered, and there were no quantitative analyses.
Illuminance (3) × color (6) interactions (this research).	For different initial emotions, LME, OLS, and MLP models were applied across 18 lighting conditions.	The study lacked multimodal data.

**Table 2:** Correlation analysis of emotional responses with light conditions.

Dependent Variable	Effect	H	df	*p*-Value
Valence	Color	4.482	5	0.482
	Illuminance	57.966	2	0.000 **
	Color * Illuminance	8.364	10	0.593
Arousal	Color	14.574	5	0.012 *
	Illuminance	40.403	2	0.000 **
	Color * Illuminance	10.386	10	0.407

Note: * *p* < 0.05; ** *p* < 0.01.

**Table 3:** LMM analysis results of physiological responses with light color and illuminance.

Physiological Variable	Light Color	Illuminance (lx)	Interaction (Color × Illuminance)	Coefficient (β)	*p*-Value
FMT (Δ)	–	20 lx		−0.043	0.035 *
	–	–	Green 180 lx	+0.076	0.009 **
	–	–	Green 20 lx	+0.071	0.015 *
	–	–	Purple 20 lx	+0.589	0.000 ***
FAA (Δ)	–	–	Purple 20 lx	+0.049	0.000 ***
TBR (Δ)	–	–	Green 180 lx	+0.520	0.040 *
LF (Δ)	Blue	–	–	+0.115	0.004 **
	Red	–	–	+0.085	0.031 *
	–	–	Blue 20 lx	+0.121	0.031 *
HF (Δ)	–	–	Red 180 lx	−0.152	0.041 *
LF/HF (Δ)	Blue	–		+0.083	0.009 **
	Purple	–		+0.712	0.044 *
SDNN (Δ)	–	–	Red 180 lx	+0.937	0.023 *
F3_γ (Δ)	–	–	Purple 20 lx	+0.056	0.028 *

Note: LMM = Linear Mixed Model; predictors include light color, illuminance, and their interaction. Physiological variables: FMT = Frontal Midline Theta; FAA = Frontal Alpha Asymmetry; TBR = Theta/Beta Ratio; LF = Low-Frequency HRV; HF = High-Frequency HRV; SDNN = Standard Deviation of NN intervals; Δ = condition difference (Last–First) * *p* < 0.05, ** *p* < 0.01; *** *p* < 0.001.

**Table 4:** Comparison of the results of emotion prediction using Random Forest.

	Valence		Arousal		Mean
	Accuracy	Recall	Accuracy	Precision	Accuracy
All	0.89	0.89	0.79	0.79	82
First 10 s	0.86	0.86	0.79	0.79	83
Last 10 s	0.82	0.82	0.72	0.72	76
Diff	0.84	0.84	0.78	0.78	81

## Data Availability

The data that support the findings of this study are available from the author upon reasonable request.

## Abbreviations

The following abbreviations are used in this manuscript:

EEG	Electroencephalography
ECG	Electrocardiography
PAD	Pleasure–Arousal–Dominance
HRV	Heart Rate Variability
LMM	Further Linear Mixed Model
LF	Low Frequency
HF	High Frequency
SDNN	Standard Deviation of NN intervals
FAA	Frontal Alpha Asymmetry
FMT	Frontal Midline Theta
TBR	Theta/Beta Ratio
LightGBM	Light Gradient Boosting Machine
XGBoost	Extreme Gradient Boosting
SVM	Support Vector Machine
Didd	Different
SEM	Structural Equation Modeling
SMOTE	Synthetic Minority Over-sampling Technique
LASSO	Least Absolute Shrinkage and Selection Operator

## Appendix A

### Appendix A.1

We compared the prediction accuracy of several models used during training, including RF, LightGBM, XGBoost, and SVM. Table A1 presents the results of this comparison for emotion prediction.

**Table A1:** Comparison of emotion prediction using different models.

	Valence (All Data)		Arousal (All Data)	
	Accuracy	Recall	Accuracy	Precision
Random Forest	0.85	0.85	0.79	0.79
LightGBM	0.58	0.58	0.63	0.63
XGBoost	0.79	0.75	0.65	0.63
SVM	0.76	0.79	0.70	0.70

### Appendix A.2

Table A2 lists the 12 pairs of emotional phrases used in the questionnaire, covering positive and negative affect as well as valence and arousal dimensions for each lighting condition.

**Table A2:** Twelve Pairs of Emotional Phrases in the Subjective Questionnaire.

Bored–Interested	Sad–Happy	Lethargic–Excited	Unpleasant–Pleasant
Angry–Satisfied	Tense–Calm	Drowsy–Focused	Low Arousal–High Arousal
Upset–Joyful	Tired–Alert	Anxious–Serene	Stressed–Contented
